# The health care utilization of people in prison and after prison release: A population-based cohort study in Ontario, Canada

**DOI:** 10.1371/journal.pone.0201592

**Published:** 2018-08-03

**Authors:** Fiona G. Kouyoumdjian, Stephanie Y. Cheng, Kinwah Fung, Aaron M. Orkin, Kathryn E. McIsaac, Claire Kendall, Lori Kiefer, Flora I. Matheson, Samantha E. Green, Stephen W. Hwang

**Affiliations:** 1 Department of Family Medicine, McMaster University, Hamilton, Canada; 2 St. Michael’s Hospital, Toronto, Canada; 3 Institute for Clinical Evaluative Sciences, Toronto, Canada; 4 Schwartz/Reisman Emergency Medicine Institute, Sinai Health System, Toronto, Canada; 5 Department of Family and Community Medicine, University of Toronto, Toronto, Canada; 6 Dalla Lana School of Public Health, University of Toronto, Toronto, Canada; 7 Research Services, Nova Scotia Health Authority, Halifax, Canada; 8 C.T. Lamont Primary Health Care Research Group, Bruyère Research Institute, Ottawa, Canada; 9 Department of Family Medicine, University of Ottawa, Ottawa, Canada; 10 Ontario Ministry of Community Safety and Correctional Services, Toronto, Canada; 11 Centre for Criminology and Sociolegal Studies, University of Toronto, Toronto, Canada; Public Health Agency of Canada, CANADA

## Abstract

**Background:**

Many people experience imprisonment each year, and this population bears a disproportionate burden of morbidity and mortality. States have an obligation to provide equitable health care in prison and to attend to care on release. Our objective was to describe health care utilization in prison and post-release for persons released from provincial prison in Ontario, Canada in 2010, and to compare health care utilization with the general population.

**Methods:**

We conducted a population-based retrospective cohort study. We included all persons released from provincial prison to the community in 2010, and age- and sex-matched general population controls. We linked identities for persons released from prison to administrative health data. We matched each person by age and sex with four general population controls. We examined ambulatory care and emergency department utilization and medical-surgical and psychiatric hospitalization, both in prison and in the three months after release to the community. We compared rates with those of the general population.

**Results:**

The rates of all types of health care utilization were significantly higher in prison and on release for people released from prison (N = 48,861) compared to general population controls (N = 195,444). Comparing those released from prison to general population controls in prison and in the 3 months after release, respectively, utilization rates were 5.3 (95% CI 5.2, 5.4) and 2.4 (95% CI 2.4, 2.5) for ambulatory care, 3.5 (95% CI 3.3, 3.7) and 5.0 (95% CI 4.9, 5.3) for emergency department utilization, 2.3 (95% CI 2.0, 2.7) and 3.2 (95% CI 2.9, 3.5) for medical-surgical hospitalization, and 21.5 (95% CI 16.7, 27.7) and 17.5 (14.4, 21.2) for psychiatric hospitalization. Comparing the time in prison to the week after release, ambulatory care use decreased from 16.0 (95% CI 15.9,16.1) to 10.7 (95% CI 10.5, 10.9) visits/person-year, emergency department use increased from 0.7 (95% CI 0.6, 0.7) to 2.6 (95% CI 2.5, 2.7) visits/person-year, and hospitalization increased from 5.4 (95% CI 4.8, 5.9) to 12.3 (95% CI 10.1, 14.6) admissions/100 person-years for medical-surgical reasons and from 8.6 (95% CI 7.9, 9.3) to 17.3 (95% CI 14.6, 20.0) admissions/100 person-years for psychiatric reasons.

**Conclusions:**

Across care types, health care utilization in prison and on release is elevated for people who experience imprisonment in Ontario, Canada. This may reflect high morbidity and suboptimal access to quality health care. Future research should identify reasons for increased use and interventions to improve care.

## Introduction

Worldwide, more than 10.3 million people are in prison at any given time [1], and an estimated 30 million people move through prisons annually [2]. This population experiences a disproportionate burden of morbidity and mortality [3, 4], which is particularly large at the time of release from prison [2, 5–8].

The United Nations Nelson Mandela Rules define States’ minimum obligations to provide equitable health care in prison and to attend to aftercare at the time of prison release [9]. Data on health care utilization may be used to indicate access to health care and population health status for this population. Several studies have determined that in prison, people have high rates of ambulatory care utilization [10–14] and hospitalization [13–16]. Research similarly shows that after release from prison, people have high rates of primary care use [17, 18], emergency department use [19–22], and hospitalization [5, 20, 21, 23], though findings regarding ambulatory care use are inconsistent [20, 21]. Several studies found that compared to use by the general population, ambulatory care use was higher in prison [10], and emergency department use and hospitalization were higher post-release [5, 22, 23]. However, most studies conducted to date have limitations that challenge their internal and external validity, including small or select samples [5, 19–21, 24], the lack of general population comparator groups [11, 12, 14, 15, 17–21, 24, 25], the use of self-reported data [15, 17–20, 25], and the use of data from over 20 years ago [11, 13]. Further, no studies have examined health care utilization both in prison and on release.

In Ontario, Canada, there is a universal, publicly funded health care system, in which the provincial government pays for physician and hospital services [26] in provincial prison as well as in the community. Access to provincial administrative health data provide a unique opportunity to comprehensively assess health care access for the population of persons who experience imprisonment, specifically in a wealthy country with a high performing health care system [26] and without the barriers to health care on prison release that may exist in countries that lack universal health insurance coverage [27].

In this study, we aimed to describe health care utilization rates for people released from provincial prison in Ontario in 2010, and to compare health care utilization with the general population.

## Methods

### Study design and setting

We conducted a retrospective cohort study of all persons released from provincial prison in Ontario, Canada in 2010 matched by age and sex with controls from the general population. Provincial prisons in Canada house persons who are admitted to prison prior to sentencing or who are sentenced to less than two years in prison, as well as persons sentenced to two years or longer prior to being transferred to a federal prison and those in temporary detention for other reasons [28]. We use the term “provincial prison” to represent all provincial correctional facilities, including jails, detention centres, and correctional centres. For Ontario residents, hospitalizations and medically necessary physician services are paid for through the public health insurance system, including in provincial prison.

### Study cohort

The Ontario Ministry of Community Safety and Correctional Services (MCSCS) provided identifying data on all adults released from provincial prison in 2010, including name, date of birth, sex, self-reported race, Ontario Health Insurance Plan (OHIP) number, and dates of admission and release and reasons for release. The MCSCS transferred these data to the Institute for Clinical Evaluative Sciences (ICES), an independent, non-profit organization funded by the Ontario Ministry of Health and Long-Term Care, which houses health administrative data for Ontario residents. We linked data on persons released from provincial prison with a unique identifier (IKN), which is an encoded OHIP number, in the Registered Persons Database (RPDB), which is a comprehensive database of all persons in Ontario who are eligible for OHIP coverage. To link data, we used the OHIP number when provided and valid, or else we used a validated deterministic or probabilistic linkage method using name (or names for those with multiple names/aliases) and date of birth [29]. We excluded linkages that seemed to be incorrect, including for persons whose date of birth or sex differed between the MCSCS data and the RPDB, if the same IKN matched to multiple persons, if MCSCS data showed that the person was in prison after the date that the person died in the RPDB, or if the person had accessed health care after the date of death in the MCSCS data.

To identify persons released to the community in 2010, the prison release group, we excluded persons who had a release period of 1 day or less in 2010 based on the assumption that such short releases represent mainly administrative status changes rather than a true release to the community and would not represent an opportunity to seek health services. For this analysis, we excluded persons whose reason for release was listed as death or transfer to federal prison, or was related to immigration, as those persons would not utilize provincially funded health care in the community after release.

For each person released from provincial prison to the community, we randomly selected four age- and sex-matched controls in the RPDB who were registered for OHIP coverage on the date of release for the person released from prison.

### Variables

#### Socio-demographic data

For each person, we accessed neighbourhood-level data on income quintile and rurality (with rural/small town defined as community <10,000 persons) using the postal code at the time of prison release. Using the MCSCS data, we examined time in provincial prison and self-reported race, which is reported by individuals on admission to custody; we maintained the category names provided by the MCSCS, *e*.*g*. “Aboriginal” for Indigenous persons. No data on race were available for general population controls.

#### Comorbidities

Using previously validated methods [30–35], we examined the proportion of persons with a diagnosis of each of the following chronic conditions ever: diabetes, hypertension, chronic obstructive pulmonary disease (COPD), asthma, and congestive heart failure (CHF), or HIV infection. We considered a person as having a diagnosis of a mental disorder if that person had one or more physician billings for that mental disorder in the past two years (S1 Protocol), including mood disorders, schizophrenia, substance-related disorders, and anxiety disorders. For each person, we used the Johns Hopkins Adjusted Clinical Group Case Mix System [36] to determine the past year number of Aggregated Diagnosis Groups (ADGs), which represent 32 diagnosis clusters that indicate the burden of disease comorbidity [37].

#### Outcome

We accessed data in the OHIP database for ambulatory care visits, the CIHI National Ambulatory Care Reporting System (NACRS) for emergency department visits, the Ontario Mental Health Reporting Systems (OMHRS) for psychiatric hospitalizations, and the CIHI-Discharge Abstracts Database (DAD) for medical-surgical hospitalizations (defined as any hospitalization for non-psychiatric reasons). We excluded emergency department visits that were planned or scheduled, and we excluded duplicate records.

### Analysis

We compared those linked and not linked using chi squared tests based on available data from the MCSCS: age, sex, self-reported race, and length of admission leading to initial release in 2010, using t tests and chi squared tests.

We followed people from the date of admission leading to the first release in 2010 for people in the prison release group or the corresponding date in general population controls. We accounted for right censoring by calculating person-time at risk until the earliest of death, loss of OHIP eligibility, re-admission to provincial prison for the prison release group, or 90 days after the date of release or the corresponding date in the general population controls. We examined health care utilization by period under study, *i*.*e*. in prison, in the 3 months after release, and specifically during days 0–6, 7–29, and 30–89 after release, and by type of health care, *i*.*e*. ambulatory care, emergency department, medical-surgical hospitalization, and psychiatric hospitalization. We calculated health care utilization rates for each health care type and period by dividing the number of encounters or admissions for each type of health care by the person-years (PYs) at risk.

We calculated rate ratios for each type of health care utilization for the prison release group compared to general population controls. We used generalized estimating equations with a negative binomial model, and we controlled for correlation due to matching. We adjusted for neighbourhood income quintile and rurality as potential confounders of the association between incarceration status and health care utilization. We considered results that were significant at a p value of 0.05 as statistically significant.

We obtained study approval from the St. Michael’s Hospital Research Ethics Board (study 15–296) and from the Hamilton Integrated Research Ethics Board (study 4422-C). While we accessed nominal data for the purposes of data linkage, we did not obtain informed consent from participants, since the study met criteria for a waiver of consent as articulated in the Canadian Tri-Council Policy Statement on Ethical Conduct for Research Involving Humans [38], and the waiver of consent was approved by the Research Ethics Board.

The full study protocol is available (S1 Protocol), as well as STROBE and RECORD checklists (see S1 Checklist) [39].

## Results

Of 53,955 persons released from provincial prison in Ontario in 2010, we achieved valid linkage for 52,546 (97.4%) (S1 Fig). For persons released for at least one day in 2010, there were significant differences between those linked and not linked to an IKN (see S1 Table), with an older median age for those linked, longer median length of stay for those linked, and different distribution of self-reported race, but no difference in sex. We excluded an additional 3,685 persons who were not released to the community in 2010. We matched each person in the prison release group with four age- and sex-matched controls, for a total of 195,444 general population controls.

Table 1 shows characteristics of persons in the prison release group and general population controls.

**Table 1:** Characteristics of persons released from provincial prison in Ontario in 2010 and general population controls.

Characteristic		Prison release group, N = 48,861	General population controls, N = 195,444
Age	Median (IQR*)	32 (24–43)	32 (24–43)
Sex	Male	42,754 (87.5%)	171,016 (87.5%)
	Female	6107 (12.5%)	24,428 (12.5%)
Self-reported race†	Missing	4,499 (9.2%)	-
	White	28,745 (58.8%)	-
	Black	5,568 (11.4%)	-
	Aboriginal	4,954 (10.1%)	-
	Other	5,095 (10.4%)	-
Neighbourhood income quintile	Missing	2,317 (4.7%)	1,009 (0.5%)
	1 (lowest)	18,151 (37.1%)	39,076 (20.0%)
	2	10,481 (21.5%)	39,113 (20.0%)
	3	7,706 (15.8%)	39,044 (20.0%)
	4	5,923 (12.1%)	39,978 (20.5%)
	5	4,283 (8.8%)	37,224 (19.0%)
Rural/Small Town	Yes	6,339 (13.0%)	20,659 (10.6%)
Time in provincial prison, median days (IQR*)	Admission leading to initial 2010 release	10 (3–52)	-
	Past 5 years	72 (12–230)	-
Persons years of follow up (persons)	In prison‡	6,685 (48,861)	26,738 (195,444)
	0–6 days post-release‡	932 (48,861)	3,745 (195,444)
	7–29 days post-release‡	2,929 (47,870)	12,299 (195,393)
	30–89 days post-release‡	6,917 (44,939)	32,037 (195,231)

*IQR = interquartile range.

†Data on race were not available for the general population.

‡Or the corresponding dates for general population controls.

Compared to general population controls, persons in the prison release group had a higher prevalence of all conditions examined, except for hypertension (see Table 2). The relative rate of mental disorders ranged from 5.23 for substance use disorders to 11.0 for schizophrenia for the prison release group compared to the general population. In the prison release group, the HIV prevalence was 3.84 times higher and the COPD prevalence was 2.20 times higher than in the general population controls. Persons in the prison release group also had a higher number of ADGs, as indicated by the median number and proportion of people with 5–9 or 10 or more ADGs.

**Table 2:** Health status of persons released from provincial prison in Ontario in 2010 and general population controls.

Health status indicator		Prison release group, N = 48,861	General population controls, N = 195,444	Relative rate (95% CI)
Number of ADGs*	Median (IQR*)	4 (2–7)	3 (1–5)	N/A
	0–4	25,383 (51.9%)	136,412 (69.8%)	0.74 (0.74, 0.75)
	5–9	17,395 (35.6%)	51,825 (26.5%)	1.34 (1.32, 1.36)
	≥10	6,083 (12.4%)	3,660 (1.9%)	3.38 (3.27, 3.49)
Chronic disease prevalence†	Diabetes	2,341 (4.8%)	8,047 (4.1%)	1.16 (1.11, 1.22)
	Hypertension	3,629 (7.4%)	17076 (8.7%)	0.85 (0.82, 0.88)
	Chronic obstructive pulmonary disease	2178 (4.5%)	3960 (2.0%)	2.20 (2.09, 2.32)
	Asthma	8011 (16.4%)	26,939 (13.8%)	1.19 (1.16, 1.22)
	Congestive heart failure	166 (0.34%)	507 (0.3%)	1.31 (1.10, 1.56)
HIV infection prevalence†		330 (0.7%)	343 (0.2%)	3.85 (3.31, 4.47)
Mental disorders prevalence†	Mood disorders	3,318 (6.8%)	1,521 (0.8%)	8.73 (8.22, 9.26)
	Schizophrenia	1,909 (3.9%)	696 (0.4%)	11.0 (10.1, 12.0)
	Anxiety disorders	3,757 (7.7%)	2,336 (1.2%)	6.43 (6.12, 6.77)
	Substance–related disorders	8,270 (16.9%)	2,392 (1.2%)	5.23 (5.10, 5.36)

*ADGs = Aggregated Diagnosis Groups, IQR = interquartile range.

†Diagnosis based on health administrative data.

In the prison release group, ambulatory care utilization and psychiatric hospitalization rates were higher in prison than in the three months after release (Table 3). In contrast, the rates of emergency department use and medical-surgical hospitalization were lower in prison than in the three months after release. Comparing the time in prison to the week after release, ambulatory care utilization decreased and the rate of emergency department utilization, medical-surgical hospitalization, and psychiatric hospitalization all increased substantially. Rates of ambulatory care utilization were lower in all time periods post-release and rates of emergency department utilization and medical-surgical hospitalization were higher in all time periods post-release compared to in prison, while psychiatric hospitalization rates decreased over the 3 months post-release, reaching rates in post-release days 30–89 that were lower than rates in prison.

**Table 3:** Health care utilization for persons released from provincial prison in Ontario in 2010 (N = 48,861) and general population controls (N = 195,444), by health care type and period relative to time in prison*.

Health care type	Period relative to time in prison*		Rate of use (95% CI)		Rate ratio† (95% CI)
			Prison release group	General population controls	
Ambulatory care (encounters/ person-year)	In prison		16.0 (15.9,16.1)	3.2 (3.2, 3.2)	5.3 (5.2, 5.4)
	Post-release	3 months	8.1 (8.0, 8.1)	3.4 (3.4, 3.4)	2.4 (2.4, 2.5)
		0–6 days	10.7 (10.5, 10.9)	3.5 (3.4, 3.5)	3.2 (3.1, 3.3)
		7–29 days	8.0 (7.9, 8.1)	3.4 (3.4, 3.5)	2.4 (2.4, 2.5)
		30–89 days	7.6 (7.5, 7.6)	3.4 (3.4, 3.4)	2.3 (2.2, 2.4)
Emergency department (encounters/ person-year)	In prison		0.7 (0.6, 0.7)	0.3 (0.3, 0.3)	3.5 (3.3, 3.7)
	Post-release	3 months	1.6 (1.6, 1.7)	0.3 (0.3, 0.3)	5.0 (4.9, 5.3)
		0–6 days	2.6 (2.5, 2.7)	0.3 (0.3, 0.4)	7.9 (7.2, 8.6)
		7–29 days	1.7 (1.6, 1.7)	0.3 (0.3, 0.4)	5.1 (4.8, 5.4)
		30–89 days	1.4 (1.4, 1.5)	0.3 (0.3, 0.4)	4.3 (4.1, 4.5)
Medical-surgical hospitalization (admissions/ 100 person-years)	In prison		5.4 (4.8, 5.9)	2.4 (2.3, 2.6)	2.3 (2.0, 2.7)
	Post-release	3 months	9.9 (9.3, 10.5)	3.1 (3.0, 3.2)	3.2 (2.9, 3.5)
		0–6 days	12.3 (10.1, 14.6)	2.4 (1.9, 2.9)	5.3 (3.9, 7.0)
		7–29 days	9.6 (8.5, 10.8)	3.4 (3.1, 3.8)	2.8 (2.4, 3.3)
		30–89 days	9.4 (8.7, 10.1)	3.0 (2.8, 3.2)	3.1 (2.8, 3.5)
Psychiatric hospitalization (admissions/ 100 person-years)	In prison		8.6 (7.9, 9.3)	0.5 (0.4, 0.6)	21.5 (16.7, 27.7)
	Post-release	3 months	7.2 (6.7, 7.7)	0.4 (0.4, 0.5)	17.5 (14.4, 21.2)
		0–6 days	17.3 (14.6, 20.0)	0.3 (0.1, 0.5)	58.3 (30.9, 110.0)
		7–29 days	8.0 (7.0, 9.0)	0.4 (0.4, 0.5)	20.5 (14.8, 28.5)
		30–89 days	5.3 (4.8, 5.9)	0.5 (0.4, 0.5)	12.1 (9.5, 15.3)

*Or the corresponding dates for general population controls.

†For the prison release group compared to general population controls, adjusted for neighbourhood income quintile and rurality.

**Fig 1:**
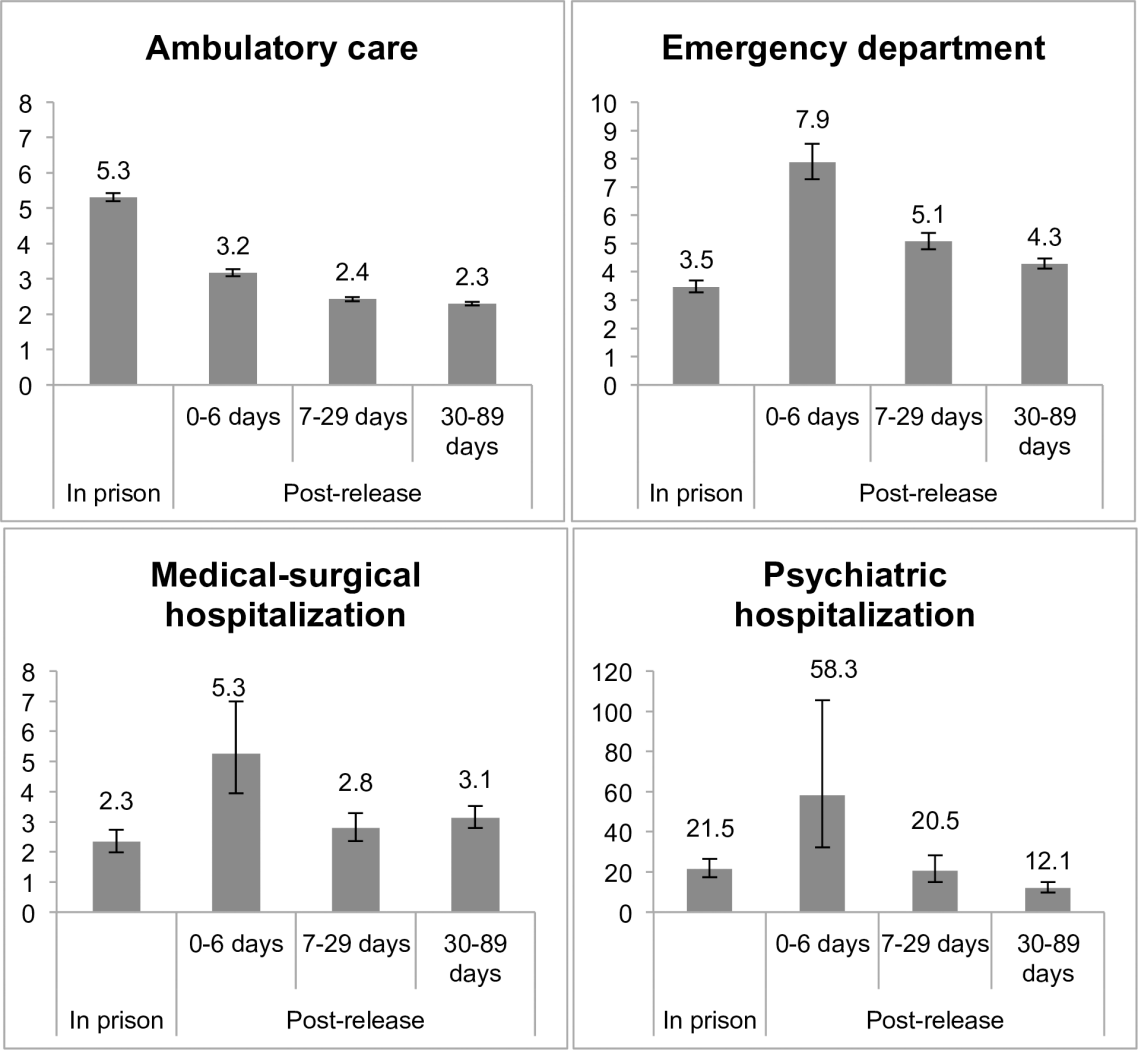
Rate ratio* of health care utilization of persons released from provincial correctional facilities in Ontario in 2010 (N = 48,861) and age- and sex-matched general population controls (N = 195,444), by health care type and period relative to time in prison† *Adjusted for neighbourhood income quintile and rurality. †Or the corresponding dates for general population controls.

Compared to general population controls, the rate of health care utilization was significantly higher for the prison release group across all time periods and types of health care, as shown in Table 3 and Fig 1.

## Discussion

In this population-based study, we found that people who experience imprisonment had significantly elevated rates of health care utilization while in prison and after release and for all types of health care examined. Rates of utilization of ambulatory care and emergency departments and of hospitalization for medical-surgical and psychiatric reasons were high compared to age- and sex-matched general population controls. There were substantial changes in rates of health care utilization at the time of release, with a large decrease in ambulatory care use and increase in emergency department use and hospitalization in the week after release.

Our study findings on health care utilization in prison are consistent with prior data on ambulatory care, which identified use rates of 6 to 20 visits per year [10, 11, 13–15], and hospitalization in Canadian and UK studies [13, 40]. Emergency department use rates in prison were several fold higher in prison than those in a 1999 Canadian study [40]. Post-release, relative rates of emergency department use and hospitalization were increased, similar to recent US studies of persons with recent criminal justice system involvement [5, 22, 41]. The increased comorbidity in the prison release group compared to the general population was also consistent with other research [3, 4].

Strengths of this study include our comprehensive assessment of health care utilization: in prison, on release, and across several types of health care. Our sample was population-based and the sample size was large. We achieved a high rate of linkage between correctional data and health administrative data, at 97.4%. Instead of relying on self-reported health care use, we accessed health administrative data, which are comprehensive for Ontario residents in the setting of universal health care. We compared rates of use with several age- and sex-matched general population controls for each person in the prison release group.

This study has several potential limitations. For health care in prison, we were not able to discern if ambulatory care visits were for administrative reasons only, or the degree to which Ministry-required physician assessments were captured in health administrative data. Similarly, on release, some persons may be obligated to participate in treatment for mental illness or substance use disorders, for example, through court-ordered drug treatment, and we were not able to distinguish care for those purposes. The data did not specify whether people were released from prison for compassionate reasons, which may be associated with health care use on release such as medical-surgical hospitalization. However, the population age distribution and the rarity of compassionate release in Ontario provincial prison make it unlikely that this would substantially affect our findings. Indigenous persons are overrepresented in provincial prisons, and health care utilization on First Nations is not captured in provincial health administrative data, which may mean that health care utilization rates post-release underestimate use; this would lead to a conservative bias. Administrative data may misclassify comorbidity status as well as socio-demographic variables. We note the substantial proportion of persons in the prison release group with missing race data (9.2%), which could bias the estimates of the proportions in each race category, and the proportion of persons with missing neighbourhood income quintile (4.7%), which is lower than expected given the frequency of homelessness at the time of prison release [42] and may therefore not accurately indicate geographic location. The prevalence of comorbidities may be inaccurate, including for mental disorders since we used diagnostic codes that have not been validated, and for HIV since provincial prisons do not routinely offer screening for bloodborne infections. We did not examine the independent effect of imprisonment on health care utilization, as this was not our study objective; a different analytic strategy would be required to explore that issue. We did not look at indicators of quality of care [43–45], as this was also beyond the scope of this study, however, this information would be relevant to understanding how well health care services in prison and in the community met general and disease-specific standards of care. The study results may not be generalizable to other jurisdictions, for example those with different health care systems or with different patterns of morbidity and mortality in prison or the general population.

Our results show that even in the context of universal health care and with high rates of ambulatory care use, rates of emergency department use and hospitalization remain very high for this population. The high rates of admission to hospital necessarily reflect the high morbidity of this population, since only people with substantial illness are admitted to hospital. It is unclear to what extent suboptimal access to quality health care may contribute to high morbidity and to emergency department use and hospitalization, for example whether people are receiving the health care they need in prison and on release to meet their needs in prison and to support continuity of care. Further work should examine ambulatory care access and quality for this population, especially in light of prior research showing that for people with chronic illness, ambulatory care may focus on specific conditions at the cost of other health conditions and preventive care [46].

A novel finding of this study is the difference in health care utilization rates in prison and post-release. Compared to use after release, the relatively high rate of ambulatory care use in prison could be appropriate for patients’ preferences and high needs, or could reflect increased need in prison due to distress related to imprisonment [10] or people not attending to their health needs after release because of competing priorities, reluctance to disclose that they were imprisoned to community-based care providers, or a lack of access to care [13]. Administrative issues may also contribute to use in prison, such as institutional requirements for a clinical assessment or to see a physician for care that is available in the community without a physician [10, 13], for example over-the-counter medications. Though ambulatory care utilization decreased substantially on release, the rates in the prison release group remained high compared to the general population, which is notable given that people face many challenges on release that may compete with attending to health needs and accessing health care, including housing access, relapse to substance use, and family issues [47–52]; this suggests people may have urgent unmet health care needs at the time of release.

Increased rates of emergency department use and hospitalization on release may reflect changes in health behaviours such as substance use, which drives high mortality on release [8, 53], worsening health status because of medication non-adherence or lack of housing on release, or a lack of adequate access to primary care [13] for routine care including prescriptions. In prison, health care providers function as gatekeepers to the emergency department, which may prevent emergency department use in prison, whether appropriately or inappropriately. Finally, hospital-based health care providers may make different decisions regarding whether to admit a patient to hospital based on whether a person is in prison or in the community, for example based on perceptions about access to monitoring and care. In particular, the substantial change in psychiatric admission rates may illustrate such differential access to care, but could also reflect acute worsening illness, for example due to medication non-adherence or substance use. Additional research is needed to understand both the reasons for high use and differences in rates of use on release, with a particular focus on continuity of care for persons with specific diseases such as mental illness, substance use disorders, HIV, and other chronic diseases, and, if indicated, evidence-based strategies should be implemented to support persons with comorbidity in achieving continuity of care [18, 21, 54–57].

The large increase in emergency department use and hospitalization on prison release indicates the need to focus on health care and health status at the time of release from prison, consistent with the obligations defined in the Nelson Mandela Rules [9]. Even in the context of a wealthy country such as Canada with universal public health insurance, we need to consider ways to change the health care system to address the needs of this population. We should advance work to improve appropriate access to and acceptability of health care for this population, building on evidence in this field [18, 21, 54–56], such as linkage with appropriate health care and social supports at the time of release from prison. Interventions should focus on health care in prison and in the community at the health system, provider, and patient levels, with explicit attention to supporting people with multi-morbidity and social complexity.

## Supporting information

S1 Checklist Completed RECORD statement. (DOCX)

S1 Protocol (DOC)

S1 Fig Flow chart for linkage of data. (DOCX)

S1 Table Comparison of characteristics of persons released for 1 or more days in 2010 who were linked and not linked to an IKN. (DOCX)
