# Supplementary material for: The health care utilization of people in prison and after prison release: A population-based cohort study in Ontario, Canada
Source: PLoS One. 2018 Aug 3;13(8):e0201592. doi: 10.1371/journal.pone.0201592 (PMC6075755; doi:10.1371/journal.pone.0201592)
Supplement: S1 Protocol — (DOC) [file pone.0201592.s002.doc]

**Research protocol:** The health care utilization of people in prison and after prison release: A population-based cohort study in Ontario, Canada

**Objective:** To describe health care utilization rates for people released from provincial prison in Ontario in 2010, and to compare health care utilization with the general population.

**Background**

Worldwide, more than 11 million people are incarcerated at any given time , and an estimated 30 million people move through the prison system annually . In Canada, there are approximately 251,629 adult admissions to provincial and territorial facilities and 8,006 to federal facilities over the course of a year , and an average of about 40,000 people in correctional facilities on any given day . In Ontario, about 50,000 people are admitted to provincial correctional facilities each year . These numbers translate into almost 1 in 200 people in Canada and in Ontario who experience detention or incarceration each year.

There are several reasons to focus on improving the health of people who experience detention and incarceration . International data indicate that the health of this population is poor compared with the general population, with a disproportionate burden of mental illness, infectious diseases, chronic diseases, and premature mortality . This burden of disease affects the general population directly through increased health care costs and through the transmission of communicable disease after people are released from detention, for example with infections such as hepatitis C, HIV, and tuberculosis. Further, an emerging literature suggests that poor health outcomes in ex-offenders may affect public safety and re-incarceration, for example in the case of inadequately treated mental illness and substance use disorders , and that better access to health care is associated with less recidivism . Finally, in Canada, provincial and territorial governments are obligated to provide universal, comprehensive, and accessible health care to eligible persons , and this obligation extends to people while in provincial or territorial custody and at the time of release from custody to the community.

Access to appropriate health care is instrumental to health, and may decrease health care costs. Health care utilization data would provide valuable information about access to care, and potentially define an area on which to focus efforts to improve health for this population. However, little is known about access to health care in people with a history of detention or incarceration in Canada, either while in custody or in the community. Data on persons in federal custody in the 1990s show high rates of health care utilization while in custody, including primary care use, emergency department use, and hospitalization . Regarding care while not in custody, a recent study with women and men in a provincial facility in Ontario found that in the year before admission, one third of participants did not have a primary care provider and participants reported a mean of 2.1 visits to the emergency department ; these number are higher than those for the general population . Studies on access to mental health care in particular have identified a wide range of rates of outpatient care but consistently high rates of psychiatric hospitalization . Limitations of these data are that they are self-reported , they were collected many years ago and therefore may not reflect current use , they represent only a small percentage of the population in custody, *i.e.* persons in a single facility in a single province or persons in federal custody, and they do not include hospitalization for all reasons. That notwithstanding, these data suggest that primary care utilization is low for this population while not in custody, and that emergency department use and hospitalization rates are high.

Two other types of information also suggest that health care utilization may not be optimized in this population. First, there is overrepresentation of characteristics associated with high rates of health care utilization , such as mental illness, substance use disorders, homelessness, and poverty . Second, recent studies from the US and the UK have found low rates of primary care access, high rates of emergency department use (including for “regular health care”) and high rates of hospitalization . In contrast, a recent study from Australia showed high rates of primary care utilization in the two years after release from custody, as well as high rates of emergency department utilization and hospitalization ; this may mean that primary care utilization alone does not necessarily lower rates of other types of health care utilization. There is also evidence from a national study in the US that quality of care in custody is substandard, as indicated by the percentage of persons that had received a medical examination at all and subsequent to serious injury, and access to lab testing for persons with relevant diseases .

The time of release from custody has been shown to be associated with particularly high rates of death and hospitalization . Since poor health status contributes to greater health care utilization , health care utilization may be especially high in this population at the time of release from custody.

In this study, we will characterize the health care utilization of persons who experience detention and incarceration in provincial correctional facilities in Ontario before admission to custody, while in custody, and after release. We will compare the health care utilization rates with rates for matched population controls.

**Methods**

*Overview*: We will link data from the Ontario Ministry of Community Safety and Correctional Services on adults released in 2010 from provincial prison with data on health care utilization from several health care databases, which are housed at the Institute for Clinical Evaluative Sciences (ICES), an independent, non-profit organization funded by the Ontario Ministry of Health and Long-Term Care. We will use these data to determine rates of health care utilization for this population and compared with the general population.

*Provincial prison group*: We estimate that 50,000 adults were released from provincial correctional facilities in 2010 : 5,000 women and 45,000 men . This includes people who were admitted to custody prior to sentencing (“detained”) and people who were sentenced to less than two years in custody (“incarcerated”). (Those sentenced to two years or longer serve their sentences in the federal system).

*Data transfer and linkage*: We will develop a data sharing agreement between the Ministry of Community Safety and Correctional Services (MCSCS), the investigators and St. Michael’s Hospital, and ICES. We will obtain the following data from the MCSCS on persons released from prison in 2010: name, date of birth, sex, home address, self-reported race, provincial health insurance number, and number and length of periods in custody in provincial facilities from 2005 to 2015, and reasons for release from custody.

We will arrange for the transfer of data on the provincial prison group from the MCSCS to ICES. Staff at ICES will link persons in the provincial prison group to individuals in the Registered Persons Database (RPDB). The RPDB is a roster of all individuals eligible for the Ontario Health Insurance Plan (OHIP). Through the RPDB, we will be able to access a unique ICES encoded health card number (IKN), with which we can identify individuals across health care databases. For persons in the provincial prison group with a known OHIP number (estimated to be 80%), we will do a deterministic linkage with the RPDB. For persons in the provincial prison group with no known OHIP number, we will do a probabilistic linkage with the RPDB based on name, sex, date of birth, and home address, using validated procedures . We will remove linkages that are likely incorrect, i.e. if the date of birth or sex differed between the MCSCS and RPDB data, if an IKN matched to multiple persons, if the MCSCS data showed a person was in prison after the RPDB date of death, or if the person remained OHIP-eligible in the RPDB after the date of death in the MCSCS dataset.

*Prison release group:* We will exclude persons not released to the community in 2010, *i.e.* those with a release period of 1 day or less, persons transferred to federal prison on release, persons who died in provincial prison, or those whose reason for release is related to immigration.

*Comparator data*: For each person in the prison release group, we will identify up to four controls who are registered in the RPDB on the date of release of each person in the provincial prison group. We will select the control by matching for age and sex, given that these factors may be associated with health care utilization .

*Health care utilization data:* Using the IKN, we will access data in the OHIP database for ambulatory care visits (primary care and non-primary specialist care), the National Ambulatory Care Reporting System (NACRS) for emergency department visits, and the CIHI-Discharge Abstracts Database (DAD) and Ontario Mental Health Reporting Systems (OMHRS) database for hospitalization data. We will include all emergency department visits except those that were planned or scheduled, and we will exclude duplicate records.

*Other variables:* We will look at baseline characteristics of persons in both groups, as defined in Appendix 1.

*Follow up period:* We will adjust the period of follow up for death, re-admission to provincial prison, loss of OHIP eligibility, or 90 days post-release.

*Analysis*: We will calculate health care utilization rates as the number of encounters for each source of health care (*i.e.* ambulatory care use, emergency department use, and hospitalization) in custody and after release, and divided by the amount of time under study. We will calculate health care use by period after release.

To compare the health care utilization of persons in the provincial prison group and matched controls, we will use generalized estimating equations with negative binomial models. These models will provide estimates of risk ratios and 95% confidence intervals for the participants in the prison release group compared with matched controls, and will control for correlation due to matching.

*Power*: The size of the prison release group is about 50,000, which is the estimated number of persons who were released from provincial custody in 2010. Having data from a full year prevents any bias that could occur due to seasonal variation in health care utilization. Based on the rates of health care access in Ontario identified in another marginalized population , we anticipate that we will have adequate power to determine the rates of ambulatory care use, emergency department use, and hospitalization with narrow confidence intervals, including for sex and age groups, for specific diseases, and in specific time intervals. There should also be adequate power for relative risk estimates.

*Limitations:* We will be able to access data on health care utilization only for persons in the provincial prison group who have an OHIP number. We estimate that this is a large proportion of the cohort, since an estimated 90% of people who are admitted to a provincial facility have an up-to-date OHIP number, and the correctional facility staff apply for an OHIP number for any persons in the remaining 10% who are eligible for coverage and who have a physician visit during their time in custody (in order to compensate the treating physician). Not being able to identify health care utilization data for some persons in the provincial prison group would result in an underestimate of the overall health care utilization for the provincial prison group. There may be a systematic bias in our data if the health care utilization rates and patterns were significantly different in persons with no OHIP card or eligibility; it is difficult to anticipate in which direction this bias would be, but any bias would likely be small given the large proportion of persons with an OHIP number at the time of release from custody.

The data available on health care utilization are relatively comprehensive. In terms of primary care data, the data at ICES for the time period under study capture an estimated 95% of primary care encounters, including fee-for-service billings, shadow billings, and Community Health Centre encounters. In prison, the initial physician visit may not be billed to OHIP, since these are third party examinations required by the Ministry of Community Safety and Correctional Services for each person admitted.

**References**

Appendix 1. Variable definitions and data sources

| **Type of variable** | **Variable** | **Definition** | **Data source** |
| --- | --- | --- | --- |
| Socio-demographic | Age | Continuous, derived categories using data from the Registered Persons Database | ICES |
|  | Race | Self-reported race, categorical variable, further categorized into Missing/White/Black/Aboriginal/Other | MCSCS |
|  | Neighbourhood income quintile | Postal codes converted into census tracts using the Postal Code Conversion File (PCCF+) from Statistics Canada. | ICES |
|  | Rurality | Postal codes converted into census tracts using the Postal Code Conversion File (PCCF+) from Statistics Canada. Rural/ small town defined as community size <10,000 people. | ICES |
| Criminal justice system involvement | Admission leading to initial 2010 release | Days in provincial prison | MCSCS |
|  | Time in provincial prison between January 1, 2005 and date of admission leading to initial release in 2010 | Days in provincial prison |  |
| Comorbidities | Diabetes | As per validated algorithm | ICES |
|  | Hypertension | As per validated algorithm | ICES |
|  | Chronic obstructive pulmonary disease | As per validated algorithm | ICES |
|  | Asthma | As per validated algorithm | ICES |
|  | Congestive heart failure | As per validated algorithm | ICES |
|  | HIV infection | As per validated algorithm | ICES |
|  | Mood disorders | ICD-10-CA: F30, F31, F32, F33, F34, F38, F39, F53.0  DSM-IV: 296.x, 300.4x, 301.13  PROVDX: 6  OHIP 296, 311 | ICES |
|  | Schizophrenia | ICD-10-CA: F20 (excluding F20.4), F22, F23, F24, F25, F28, F29, F53.1  DSM-IV: 295.x, 297.x, 298.x  PROVDX: 5  OHIP 295, 297. 298 | ICES |
|  | Anxiety disorders | ICD-10-CA: F40, F41, F42, F43, F48.8, F48.9  DSM-IV: 300, 300.0x, 300.2x, 300.3x, 308.3x, 309.0x, 309.24, 309.28, 309.4x, 309.8x, 309.9x  PROVDX: 7, 15  OHIP 300, 309 | ICES |
|  | Substance–related disorders | ICD-10-CA: F55, F10-F19  DSM-IV: 291.x (excluding 291.82), 292.x (excluding 292.85), 303.x, 304.x, 305.x  PROVDX: 4  OHIP 303, 304 | ICES |
|  | Aggregated diagnosis groups | Generated using the Johns Hopkins Adjusted Clinical Group (ACG) Case-Mix System |  |
| Health care utilization | Primary care | OHIP data: location = OFFICE, laboratory records and claims from nonmedical practitioners excluded, claims by the same physician for the same patient on the same day to be a single health care encounter, specialty = 00 (GP/FP) | ICES |
|  | Ambulatory care | OHIP data: location = OFFICE, laboratory records and claims from nonmedical practitioners excluded, claims by the same physician for the same patient on the same day to be a single health care encounter, specialty ^= 00 |  |
|  | Emergency department visits | NACRS: all visits, excluding planned or scheduled visits | ICES |
|  | Psychiatric hospitalization | OMHRS: Any hospitalization for psychiatric reasons | ICES |
|  | Medical-surgical hospitalization | DAD: Any non-psychiatric hospitalization | ICES |
