# Supplementary material for: The health care utilization of people in prison and after prison release: A population-based cohort study in Ontario, Canada
Source: PLoS One. 2018 Aug 3;13(8):e0201592. doi: 10.1371/journal.pone.0201592 (PMC6075755; doi:10.1371/journal.pone.0201592)
Supplement: S1 Fig — (DOCX) [file pone.0201592.s003.docx]

**Supplementary Figure 1. Flow chart for linkage of data**

Persons released from provincial prison in Ontario in 2010:

53,955 persons

Direct, deterministic, or probabilistic linkage with ≥1 valid IKN:
53,331 persons, 60,785 person-IKN linkages

Valid linkage:

52,546 persons, 58,363 person-IKN linkages

Excluded linkages that were likely incorrect:

- date of birth different in MCSCS and RPDB data (n=1,569)
- sex different in MCSCS and RPDB data (n=200)
- IKN matched to multiple persons (n=572)
- MCSCS data showed the person was in prison after the date of death in the RPDB (n=45)
- RPDB showed the person was OHIP-eligible after the date of death in MCSCS data (n=36)

Excluded persons not released to community in 2010:

- release period of ≤ 1 day (n=233)
- transfer to federal prison on release (n=2,178)
- died in provincial prison (n=7)
- reason for release related to immigration (n=1,267)

Prison release group:

48,861 persons
