# Supplementary material for: The health care utilization of people in prison and after prison release: A population-based cohort study in Ontario, Canada
Source: PLoS One. 2018 Aug 3;13(8):e0201592. doi: 10.1371/journal.pone.0201592 (PMC6075755; doi:10.1371/journal.pone.0201592)
Supplement: S1 Table — (DOCX) [file pone.0201592.s004.docx]

**Supplementary Table 1. Comparison of characteristics of persons released for 1 or more days in 2010 who were linked and not linked to an IKN**

| Characteristic | | Unlinked (N=1,396) | Linked (N=52,313) | p value |
| --- | --- | --- | --- | --- |
| Age on initial release in 2010 | Median (IQR) | 30 (23-41) | 32 (24-43) | <.001 |
| Sex, % | Female | 186 (13.3%) | 6,357 (12.2%) | 0.186 |
|  | Male | 1,210 (86.7%) | 45,956 (87.8%) |  |
| Self-reported race, % | Missing | 283 (20.3%) | 5,243 (10.0%) | <.001 |
|  | White | 612 (43.8%) | 30,318 (58.0%) |  |
|  | Black | 178 (12.8%) | 6,351 (12.1%) |  |
|  | Aboriginal | 136 (9.7%) | 5,116 (9.8%) |  |
|  | Other | 187 (13.4%) | 5,265 (10.1%) |  |
| Length of admission leading to initial release in 2010 | Median days (IQR) | 4 (1-28) | 10 (2-57) | <.001 |
